# Switchable resolution in soft x-ray tomography of single cells

**DOI:** 10.1371/journal.pone.0227601

**Published:** 2020-01-24

**Authors:** Venera Weinhardt, Jian-Hua Chen, Axel A. Ekman, Jessica Guo, Soumya G. Remesh, Michal Hammel, Gerry McDermott, Weilun Chao, Sharon Oh, Mark A. Le Gros, Carolyn A. Larabell

**Affiliations:** 1 Molecular Biophysics and Integrated Bioimaging Division, Lawrence Berkeley National Laboratory, Berkeley, California, United States of America; 2 Department of Anatomy, University of California San Francisco, San Francisco, California, United States of America; 3 Center for X-ray Optics, Material Science Division, Lawrence Berkeley National Laboratory, Berkeley, California, United States of America; Nicolaus Copernicus University, POLAND

## Abstract

The diversity of living cells, in both size and internal complexity, calls for imaging methods with adaptable spatial resolution. Soft x-ray tomography (SXT) is a three-dimensional imaging technique ideally suited to visualizing and quantifying the internal organization of single cells of varying sizes in a near-native state. The achievable resolution of the soft x-ray microscope is largely determined by the objective lens, but switching between objectives is extremely time-consuming and typically undertaken only during microscope maintenance procedures. Since the resolution of the optic is inversely proportional to the depth of focus, an optic capable of imaging the thickest cells is routinely selected. This unnecessarily limits the achievable resolution in smaller cells and eliminates the ability to obtain high-resolution images of regions of interest in larger cells. Here, we describe developments to overcome this shortfall and allow selection of microscope optics best suited to the specimen characteristics and data requirements. We demonstrate that switchable objective capability advances the flexibility of SXT to enable imaging cells ranging in size from bacteria to yeast and mammalian cells without physically modifying the microscope, and we demonstrate the use of this technology to image the same specimen with both optics.

## Introduction

There is a remarkable structural and functional diversity of biological cells. For example, the human body contains approximately 10^13^ cells, of which there are thought to be more than four hundred distinct cell types [1, 2]. Each cell type performs a specific range of functions, and within each cell, sub-structures generate particular environments optimized for certain chemical reactions. The compartmentalization of functions means we cannot simply treat a cell as a homogeneous ‘black box’. We need to build a more detailed structural-functional description.

Understanding how a cell functions requires us to develop a template of its structural makeup, and to determine the structural changes that occur as a response to environmental and genetic factors, changes in physiological or pathological states, or even just progression through the cell cycle. Since cells are so diverse in their size and shape, an equally diverse range of imaging methods is needed. As a result, light-, electron- and x-ray microscopy techniques have been developed and deployed to great success [3–6]. Different illumination sources provide unique insights and views of a cell, typically by visualizing cells with disparate contrast mechanisms [7–9] or by generating data at another level of resolution. Among the available methods, bright-field light microscopy is the most straightforward and most widely applicable [10, 11]. A great strength of light microscopes is the ability to easily switch the objective lens from low magnification to high, depending on the experimental needs. Typically, a bright-field light microscope is equipped with multiple objectives providing magnifications of between 10x up to 1000x [12]. By using a low magnification objective, a light microscope can image very large cells (albeit at low spatial resolution). Conversely, by switching to a high magnification objective, a light microscope produces images with a smaller field of view, but with much better spatial resolution. Despite the need for this degree of flexibility in any microscopy method, switching of objective lenses in soft x-ray microscopes is not simple or routine. Typically, it is time consuming, requiring breaking the vacuum, and therefore performed as a part of the microscope maintenance procedures. Consequently, one objective capable of in-focus imaging of all specimens, regardless of size, is selected. Here, we describe a method that allows specimens to be imaged by simply and quickly changing to the objective of choice in the soft x-ray microscope.

Soft x-ray tomography (SXT) has emerged as a unique method for three-dimensional (3D) imaging of whole single cells [13–16]. Soft x-rays from 284 eV to 543 eV (2.34nm to 4.4 nm), generate contrast for cells in their native, wet state without the use of chemical fixation or staining. In this so-called ‘water window’ energy range, absorption of photons by molecular building blocks, in particular carbon and nitrogen, prevails over nearly transparent water. This absorption contrast within intact hydrated cells is quantitative and is used to differentiate cellular organelles, structures and protein concentrations [17–20].

Such global 3D imaging of internal cell morphology with light and electron microscopy remains a challenge. Light microscopy techniques, such as diffraction and optical tomography [21, 22] provide only sparse resolution without full information on the organelle composition. Fluorescence microscopy excels at localizing specific proteins of interest but, even with the use of multiple fluorescent labels [23, 24], the remaining intracellular structures remain invisible. Electron microscopy (EM) can provide full structural information without labeling and achieves two orders of magnitude better spatial resolution than is obtainable with light microscopes [25]. Yet despite advances in EM’s capacity for volumetric imaging [26], it remains extremely time-consuming, which limits the ability to observe structural changes in statistically significant numbers of whole cells. Details of the differences between light, electron and x-ray microscopy of single cells has been discussed previously [27].

Typically, the spatial resolution of SXT has ranged between 25 nm to 60 nm [14, 28, 29] and is applicable to cells that range in size from 500 nm up to 15 um thick. As in light microscopy, the spatial resolution of SXT is determined by the numerical aperture of the objective. For soft x-rays, the objective lens is a circular grating (Fresnel zone plate hereafter referred to as micro zone plate or MZP) with varying line width, where the numerical aperture is governed by the width of the outermost ring. Due to challenges in fabrication of zone plates with structures smaller than 10 nm, the spatial resolution of SXT microscopy has not yet reached the diffraction limit, corresponding to the wavelength of soft x-rays used for biology (2.4 nm, 512 eV). Efforts to date have demonstrated that zone plates can be produced with a spatial resolution as high as 10 nm and 15 nm using test patterns [30, 31]. SXT imaging of biological materials has been demonstrated with 36 nm (Rayleigh resolution) for 3D imaging of thin regions of adherent cells [28, 32].

Here, we demonstrate the first step towards a flexible, multiple resolution approach with SXT. Like in light microscopes, we have equipped the x-ray microscope with a set of two objective lenses to make it easily switchable between resolutions of 60 nm and 35 nm (other combinations are possible). We chose a 60 nm objective as it has been widely applied for imaging of cells up to 15 μm × 15 μm size with the entire cell in focus [33]. As an increase in spatial resolution leads to smaller field of view, we chose a 35 nm objective, which corresponds to a 8 μm × 8 μm field of view, for higher resolution imaging. Such a field of view is still sufficient to fully accommodate other small eukaryotic cells. We describe the development of a dual zone plate imaging technology that increases the flexibility and imaging capabilities of a soft x-ray microscope to image a broad range of biological specimens.

## Materials and methods

### X-ray objectives and test patterns

**Fig 1:**
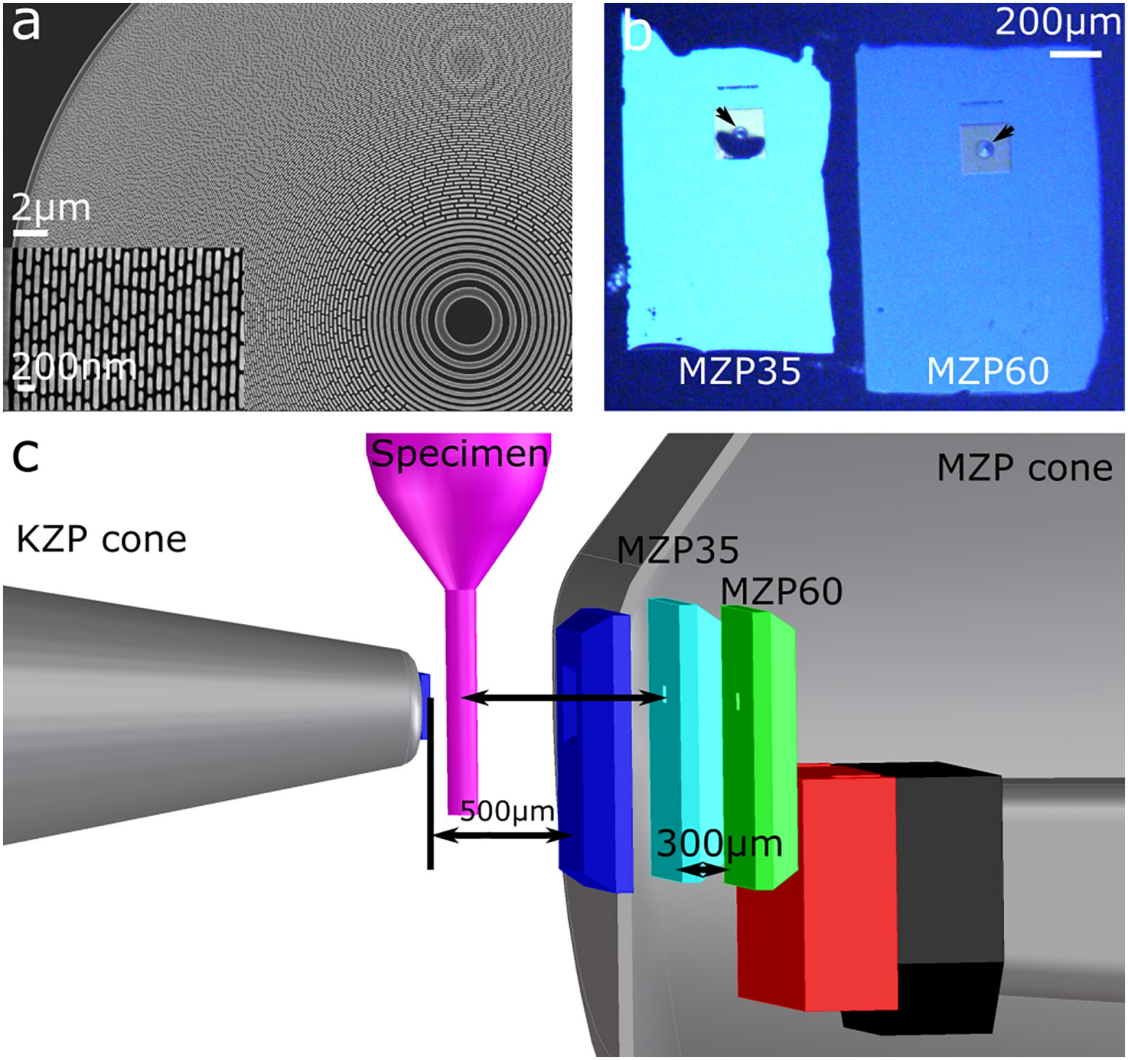
X-ray optics. (a) Scanning electron microscope images of the MZP with 35 nm outermost zone width. (b) Image of mounted MZPs acquired in reflection mode of the visible light microscope positioned the along optical axis at XM-2. The dark blue region below 35 nm MZP arises from a knickpoint in the supporting membrane. The arrows show the location of the MZPs. (c) Main optical components and their respective distances in a 3D mechanical drawing of XM-2: KZP cone—condenser zone plate located inside the steel cone; dark blue—pinholes before and after the specimen; pink—capillary with specimens; cyan and green—MZPs; red—the magnetic holder for MZPs; black—the magnetic holder positioned at the stage; MZP cone denotes the steel cone containing MZPs.

A set of micro zone plates and test patterns with varying line spacing was fabricated by conventional electron beam lithography on a 100 keV Nanowriter developed by Center for X-ray Optics at the Lawrence Berkeley National Laboratory [34]. The quality of fabricated MZPs and test patterns was confirmed on a few randomly selected areas by scanning electron microscopy. To assure that the MZPs are free of any remaining photoresist and dust, all MZPs were additionally imaged with visible light microscopy. The best set of 60 nm and 35 nm MZPs has been chosen based on both imaging methods. For the MZP with 35 nm outermost width, a piece of double sided polished silicon wafer about 3 mm × 3 mm and 300 μm in thickness was glued on the back side by UV curable epoxy glue. This piece acts as the spacer to minimize the difference in focal length between both MZPs from 925 μm without the spacer to about 625 μm. Both MZPs were then glued to a magnetic holder (see Fig 1(c) in red) by a thin layer of silicon. Due to the spacer the 35 nm MZP was closer to the specimen by 300 μm. The MZPs on the magnetic holder were mounted to its counterpart (see Fig 1(c) in black) positioned at the motorized stage for the objectives in the x-ray microscope. The relative distances between two objectives were then measured by visible light microscope positioned in-line with the x-ray path (see Fig 1(b)). The relative position was then refined by x-ray projection imaging and recorded for automatic switching. The test patterns with varying line spacing (35 nm; 40 nm; 50 nm; 60 nm) were glued to the tip of a glass capillary, aligned with respect to the optics axis and imaged in 2D with SXT.

### Specimens preparation

*E. coli* strain MG1655 was grown in 5 ml of Luria-Bertani medium at 37°C until an optical density at 600 nm of 0.1 was attained for the log phase. Yeast, *S. pombe*, was grown in the YPD medium (1% yeast extract, 2% Bacto^™^Peptone, 2% glucose) with rotary shaking at 25°C until an optical density at 600 nm of 0.5 was attained, corresponding to mid-log phase. The human B lymphocytes (GM12878) were purchased from the NGIMS Human Genetics Cell Repository, Cornell Institute of Medical Research (Camden, NJ). The B cells were maintained as suspension cultures at 37μm in Advanced RPMI-1640 medium supplemented with 15% of fetal bovine serum (FBS), L-glutamine, (Gibco™, Carlsbad, CA), and 5% CO_2_. The medium was refreshed every two to three days to maintain a cell density of (0.2 to 1) × 10^6^ ml^−1^.

All specimens were centrifuged to form a pellet and then loaded into a 6μm diameter capillary with a conventional micro-loader. Specimens were briefly examined under a light microscope, then cryo-fixed by rapid plunging into liquid propane and stored in liquid nitrogen until imaged with soft x-ray microscopy. The detailed protocol on sample preparation was published elsewhere [35].

### Imaging

The resolution test patterns were imaged with SXT set up for 1 keV at room temperature. For each objective lens, a set of 30 through-focus x-ray projection images was acquired with a step size of 1 μm. Additionally, 10 reference x-ray images were acquired without a specimen. Each x-ray image was taken with a 150 ms exposure time. A set of through-focus acquisitions assured that x-ray projection is acquired with 2D test pattern located in the center of the point spread function. The sharpest x-ray projection images were reference corrected and used for comparative analysis. Capillaries with different cell types were maintained at cryogenic temperatures (below −160°C) during imaging with a stream of liquid nitrogen-cooled helium gas [36]. All cell types were measured at 512 eV with the monochromaticity of 300. Each data set contained 92 projection images collected sequentially around the rotation axis in 3 degrees steps. For each objective, a set of 92 projections and 10 reference (flat field) images was acquired over 360 degrees of specimen rotation. The exposure time was set to 150 ms for the 60 nm objective and 400 ms for objective with 35 nm outermost zone widths. This increase in exposure time compensates 1.7^2^ x decrease in pixel area and thus total number of photons. Full 3D tomography acquisition varied from 3 to 10 minutes per specimen. Glass capillaries used for specimen handling are the most absorbing material and are first affected by x-ray radiation dose [37]. As all specimens are positioned in capillaries of the same glass thickness, we used the same exposure time for all specimens. The effective voxel size in SXT tomograms is 32 nm and 17.8 nm respectively. To acquire tomography of whole yeast and human B cell, the specimens were imaged multiple times while vertically translating specimens. For yeast we acquired 2 tomograms, for human B cell 4 tomograms, both with vertical shift of 13 μm.

### Reconstruction and data analysis

Projection images for all specimens (test patterns and cells) were normalized with reference images. For 3D reconstructions, the projection images of all cells were automatically aligned and reconstructed within 10 minutes using iterative reconstruction methods in the software package AREC3D [38]. As absorption of x-rays adheres to Beer-Lambert’s Law, 3D reconstructions are quantitative with measured x-ray attenuation in each voxel. For comparison, all data was transferred to linear attenuation coefficient in 1/μm by accounting for the pixel size. For the 60 nm objective with pixel size of 17.8 nm all data was multiplied by factor of 28.1 (the images are binned by 2, i.e. 1000/(17.8 × 2) and for the 35 nm objective by factor 47.8 correspondingly.

Yeast and the human B cell were segmented manually with Amira 6.3 based on previously established LAC values [19]. At first, we manually segmented cytoplasm and nucleus in every 20th virtual section. To obtain full 3D segmentation the label fields were interpolated and smoothed. In the nucleus, nucleolus was manually outlined based on higher LAC values corresponding to packed DNA [17]. In the cytoplasm, lipids, lysosomes, vacuoles and mitochondria were segmented semi-automatically by selection of voxels and automatic merging of neighbouring voxels with similar LAC by ‘magic wand’ option in Amira 6.3. Endoplasmic reticulum was segmented manually by outlining line features in each virtual slice.

The figures were prepared with Inkscape 0.92.4 software, 3D renderings of reconstructions were generated with Amira 6.3 (ThermoFisher Scientific).

## Results

### Instrumentation and implementation

The soft x-ray microscope used for these studies, operated by the National Center for X-ray Tomography (NCXT), is dedicated to imaging cells and their sub-cellular organization at cryogenic temperatures. This microscope, commonly known as XM-2, is located at the Advanced Light Source of Lawrence Berkeley National Laboratory (LBNL) on beamline 2.1 and was the first soft x-ray microscope designed and built specifically for biological imaging [36]. Over the last decade, XM-2 has operated with objective lenses that have outermost, and therefore resolution-defining, zone widths of 50 nm and 60 nm. The depth of focus of these lenses, used on XM-2, enables imaging cells up to 15 μm thick. However, advances in nanofabrication at the Center for X-ray Optics (CXRO) at LBNL have pushed the limits of manufacturing of our micro zone plates (MZP) to an outer zone width of better than 15 nm [39].

To enhance the imaging capabilities of XM-2, and to enable better resolution imaging of smaller specimens and regions of interest in thicker specimens, we selected an MZP objective lens with 35 nm outermost width. This lens, fabricated by CXRO, has a diameter of 59.8 μm and a focal length of 875 μm [34, 40]. The increased spatial resolution of this zone plate, when used in XM-2, results in a reduced field of view of 8 μm × 8 μm. The MZP with 60 nm spatial resolution used for the experiments described here has the same parameters as previously used objectives [19].

In Fig 1(a), a scanning electron micrograph shows the placement of the fabricated opaque zones of 35 nm width. To enable image acquisitions using either MZP for the same specimen, we mounted the 60 and 35 nm MZPs side-by-side on an in-vacuum motorized stage that translates the lenses normal to the optical axis of the microscope (Fig 1(b)). The range of motion of the translation stage is greater than the distance separating the zone plate centers [41]. To accommodate the shorter working distance of the 35 nm objective, and to reduce the possibility of a collision between back vacuum window and MZP lens mount, the 35 nm MZP was offset closer to the specimen along the optical axis by a mounted 300 μm spacer (Fig 1(c)). This side-by-side arrangement of the objective lenses enables rapid lens switching during imaging, eliminates the need to vent the vacuum chamber during microscope operation setup, and minimizes the potential risk of contamination to an expensive microscope.

We measured the stage positions required to focus each MZP on the specimen. These data were incorporated into the acquisition GUI to allow effortless switching between lenses. The change from 60 nm to 35 nm MZP leads to a 1.7 times reduction in effective pixel size and thus calls for adjustment of the CCD distance scale. This difference between the recorded image size for the two objective lenses was incorporated into the microscope control and alignment code [38].

### Characterization of objective lenses

**Fig 2:**
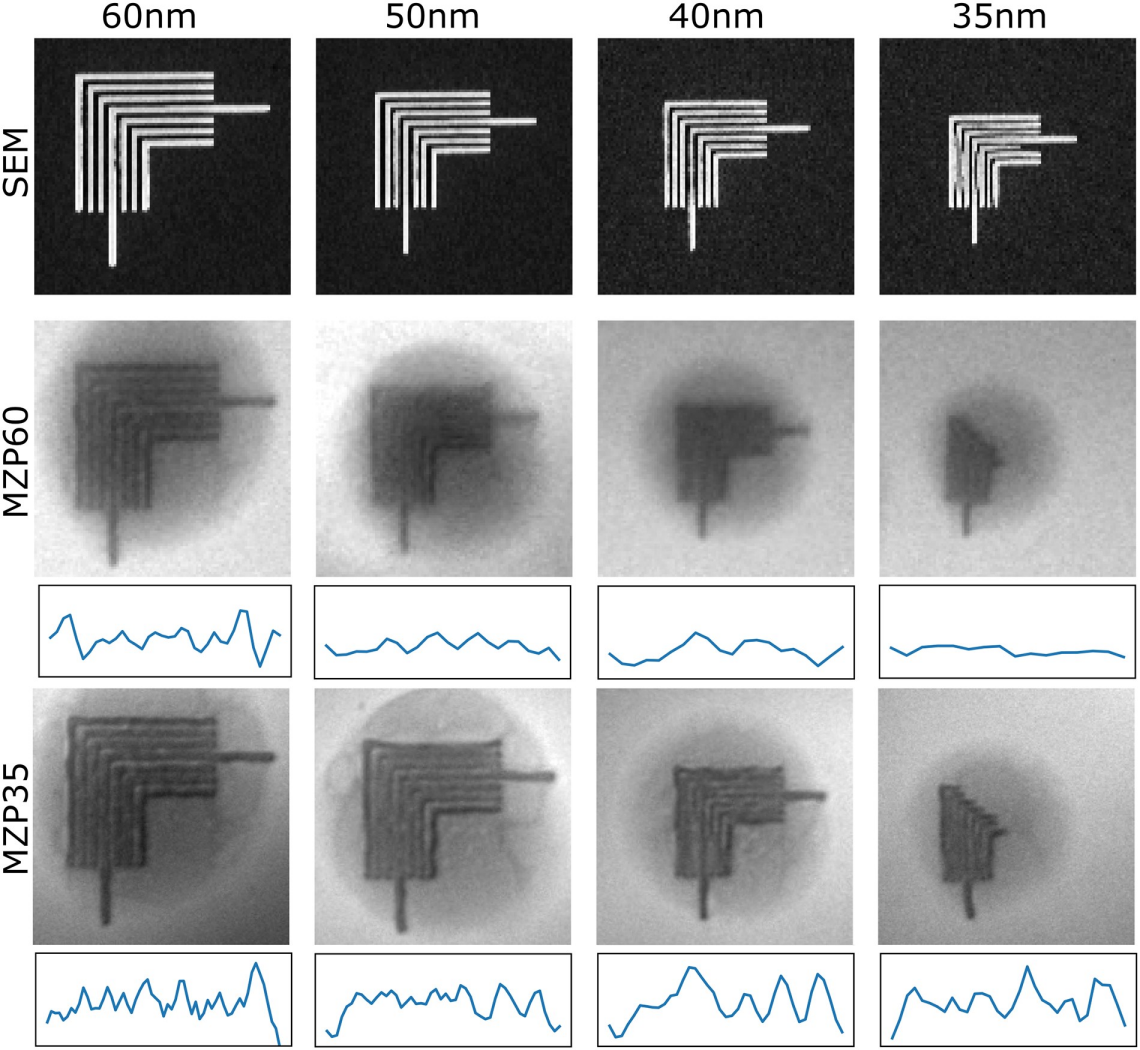
Characterization of 60 nm and 35 nm zone width objectives. Characterization of SXT objectives 60 nm and 35 nm outermost zone width. Top: SEM images of the fabricated test patterns, selected randomly on the supporting membrane. Middle: X-ray projection images of test patterns with 60 nm MZP, and bottom: the same structures visualized with 35 nm MZP. The field of view of x-ray projection images is 1.4 μm x1.4 μm. Below each x-ray projection image is a line profile through the test pattern. A spherical background on test patterns is due to remaining photoresist.

To confirm the spatial resolution of the objective zone plates, we imaged two-dimensional resolution test patterns. As with the zone plates, the test patterns were fabricated by electron beam lithography with periodic line patterns of 3560 nm periods. The dimensions and quality of fabricated test patterns were verified by SEM on randomly selected samples (Fig 2 top). X-ray projection images of test patterns with varying line spacing were collected with both objective lenses (Fig 2 middle). For the 60 nm MZP a clear decrease in spatial resolution is visible for line spacing below 60 nm. With the higher resolution 35 nm MZP, all the lines of the test pattern with 35 nm line spacing were clearly visible. This image also revealed waviness and some missing line structures in the test patterns, particularly for 35 nm line spacing, indicating defects in fabrication of this particular set of test patterns. The line profiles through the test patterns imaged with both MZPs clearly show reduced contrast for structure size below the outermost zone width of the respective lens. Fabrication of optical components with less than 35 nm line spacing has been demonstrated, but this requires more complex lithography methods, such as a double patterning technique [39]. These optics will be included in future imaging experiments using the developments described in this manuscript.

### Imaging of bacteria, yeast and eukaryotic cells

We further applied the switchable objectives to cells of varying size and complexity, specifically the bacteria *Escherichia coli* (*E. coli*), the yeast *Saccharomyces pombe* (*S. pombe*), and human lymphocytes (GM12878 cell line, B cells). All specimens were loaded into thin-walled glass capillaries, immediately cryopreserved, and stored in liquid nitrogen prior to imaging in the soft x-ray microscope.

**Fig 3:**
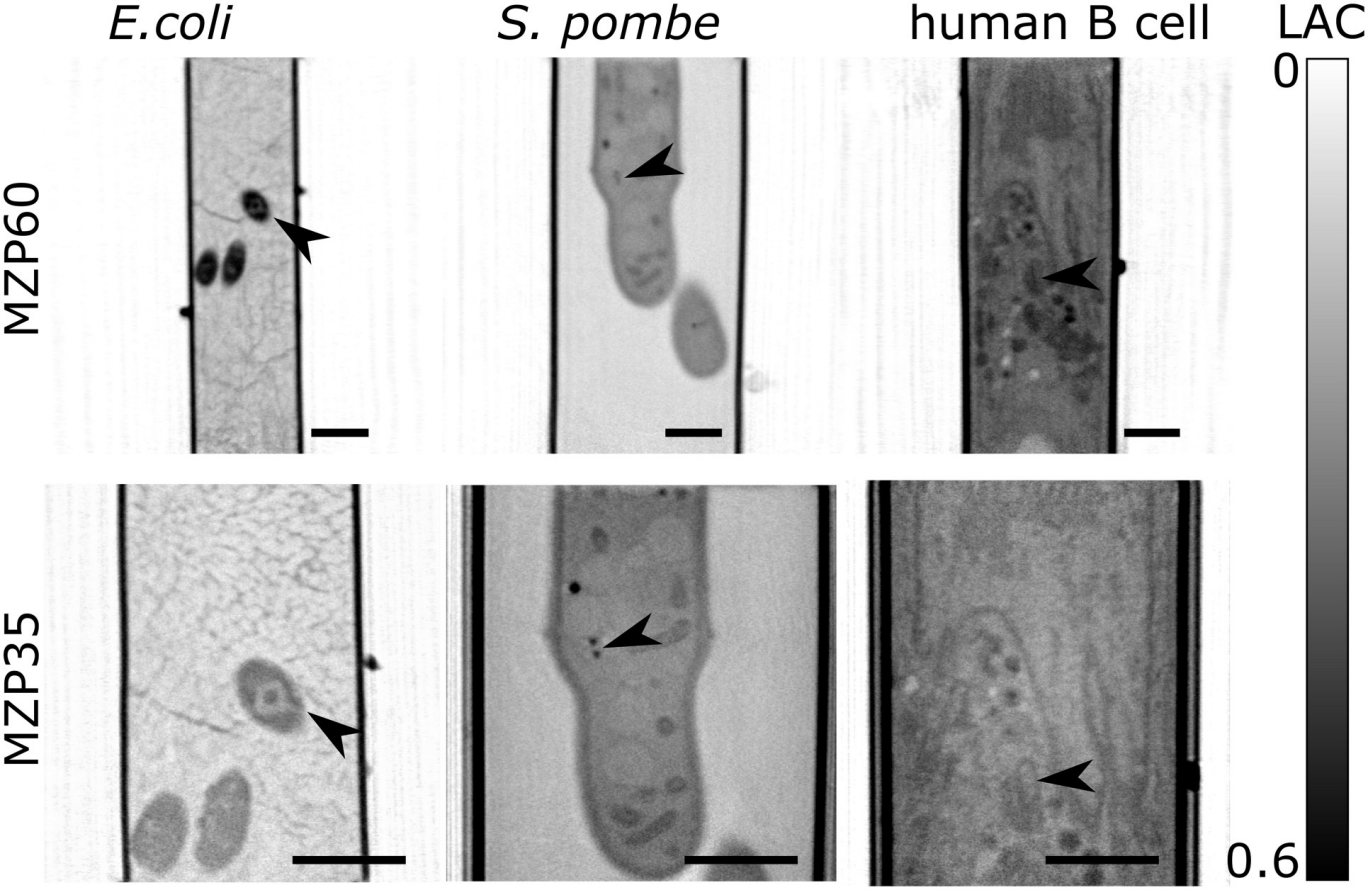
Switchable objectives applicable to diverse cells. Coronal slices from soft x-ray tomography of *E. coli*, yeast *S. pombe* and human B cell. Each specimen was imaged with both objectives at XM-2. The scale bar is 1 μm. The arrows show the same features in both datasets. The colorbar illustrates the difference in linear absorption coefficient from 0 μm^−1^ to 0.6 μm^−1^.

We imaged all cell types with both objectives by automatic switching lenses (Fig 3). The switch to between 60 nm and 35 nm MZP lenses results in a decrease in pixel size from 32 to 17.8 nm and a smaller field of view. To retain similar signal-to-noise ratios, the exposure time was increased, respectively with the pixel area from 150 ms to 400 ms respectively. The increase in exposure time with the 35 nm MZP and additional acquisition with the second objective imposed a higher radiation dose to the specimen. To minimize the impact of x-ray radiation dose [42], we collected fewer projection images, i.e. 92, and distributed the x-ray projection images evenly over 360 degrees, generating a radially symmetric dose distribution. The glass capillaries used for sample handling have been shown to accumulate a higher local dose than the specimens within, with visible wall distortions if the radiation dose is too high [37]. We did not observe such structural changes in glass shape or cellular morphology after two acquisitions for the data shown. More details on sample preparation, image acquisition and reconstructions can be found in ‘Methods’.

In comparison to the 60 nm data, an increase in sharpness is clearly visible in reconstructions of the 35 nm data. This is true for all three cell types imaged (Fig 3). Due to its relatively small size, multiple *E. coli* can be visualized simultaneously even with 35 nm objective lens. With the increased spatial resolution, the distinction between the cell wall and bacterial chromatin is apparent. The increased information is even more apparent for the yeast, *S. pombe*, where lipid droplets can clearly be distinguished as two separate droplets with the 35 nm objective. The internal structures of mitochondria and nuclear membrane are resolved in yeast, for the first time, using XM-2. Similar to yeast, the substructure of individual organelles, particularly mitochondria and lysosomes, was seen in the 35 nm reconstructions of a human B cell.

**Fig 4:**
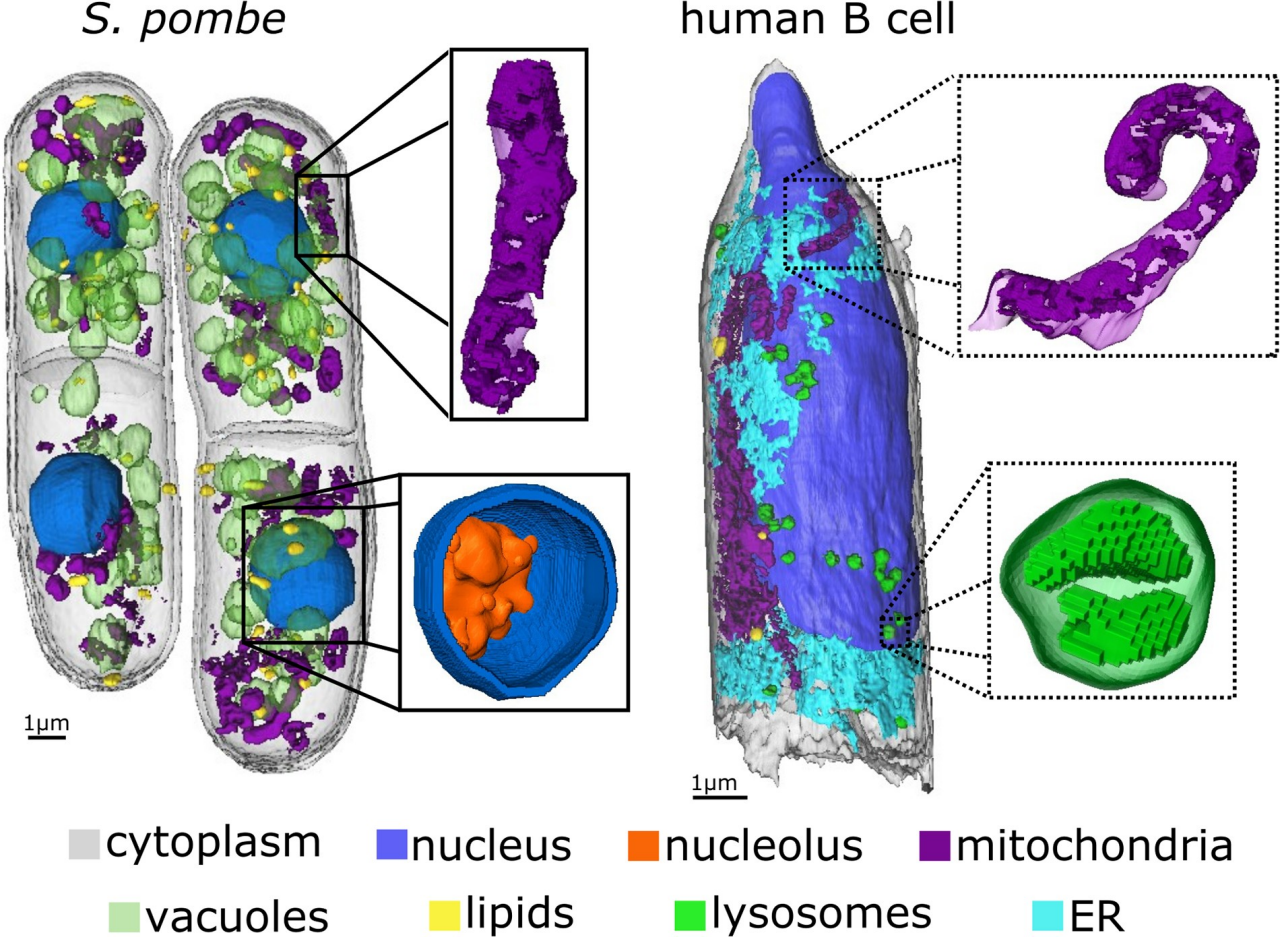
3D view with higher resolution objective. 3D rendering of all organelles imaged in yeast and human B cells with 35 nm MZP. Structural details of nuclear membrane and mitochondria in yeast together with lysosomes and mitochondria in a human B cell are shown in the respective regions. ER-endoplasmic reticulum.

The 3D renderings, segmented to show individual organelles of *S. pombe* and human B cells as seen with the 35 nm objective, are shown in Fig 4. The nucleus, the organelle encompassing the chromosomes, is clearly seen in both cell types and the nuclear envelope, as resolved by SXT, varies in thickness between 35 nm and 52 nm. While mitochondria are frequently observed by SXT, visualizing the internal structures required a spatial resolution better than 60 nm. The mitochondria found in yeast were largely interconnected tubes about 200 nm in diameter, with only a few isolated spherical mitochondria. In a few mitochondria, the membranes of folded cristae could be seen within. The mitochondria of the human B cell are also highly interconnected and tubular, although larger than those in yeast with a typical diameter of almost 500 nm. The cristae could also be resolved in individual mitochondria of the human B cell. Lipids and lysosomes are both highly x-ray absorbing spherical organelles not easily distinguished with the 60 nm objective. With the 35 nm optic, however, we were able to clearly resolve dense inclusions and internal vacuoles within the lysosomes, whereas the contents of lipid droplets appear homogenous. The typical size of a lysosome was found to be 500 nm in diameter, while lipid diameter was variable.

While there is an obvious gain in structural details visualized with the 35 nm lens, for all cell types, the imaging of cells larger than 10 μm in diameter with a high-resolution objective is difficult. To fully profit from switchable objective lenses in SXT of these large cells, we need to adopt other acquisition methods, such as local [43] and half-acquisition tomography [44]. While local tomography techniques would enable a high-resolution reconstruction of small regions of interest within a specimen, half-acquisition tomography would enable high-resolution imaging with a 2x larger field of view of an entire cell. Due to the increase in x-ray radiation dose that would result, the feasibility of these acquisition schemes needs to be investigated [44].

## Discussion

In this study, we describe developments that enable changing optics, and therefore resolution, of soft x-ray microscopes during the process of imaging cells. This capability greatly increases the flexibility of soft x-ray microscopy, which to date was limited to using a single optic that had been installed during a maintenance procedure, to image all cells. To do this we installed a motorized stage holding two adjacent objective lenses that are easily switchable under normal operating conditions. This apparatus, which is analogous to the objective turret of a light microscope, makes it possible to image a large cell with a low-resolution lens (and, therefore, extended depth of focus) and switch to a higher resolution optic for a region of interest. It also provides the flexibility needed in a microscope imaging center to accommodate the variety of cell types typically examined without a time-consuming instrument modification.

Light and electron microscopes have a much longer history of imaging biological material than do soft x-ray microscopes and, therefore, have long been equipped with sophisticated technology for changing optics. Biological soft x-ray microscopy has been hampered by the need to select one optic during a maintenance procedure and use it to image all cells at the same resolution, regardless of cell size or the type of information sought. Since one of the powerful attributes of an x-ray microscope is the ability to image thick specimens in the native state, we have typically used an optic having a large depth of focus and, therefore, lower resolution. With 50 and 60 nm optics we have been able to image large eukaryotic cells and obtain global, 3D views of the distribution of all organelles within the entire cell. However, this has meant compromising the resolution that could be achieved when imaging smaller cells such as yeast and bacteria. It has also prevented zooming in on a region of interest with a better resolution optic to obtain more detailed information in larger cells. The developments we describe here enable us, for the first time, to select the optic that best addresses the cell type being imaged and the questions being asked.

While the resolution achieved with a microscope is an important measurement of its capabilities, the information of interest to the investigator is equally as important when considering the instrument and optics to be used for data collection. With the soft x-ray microscope, it is advantageous to use a lower resolution objective lens for larger cells for those scientific questions that require global views of the cell rather than details of the internal morphology of small sub-cellular features. The higher resolution objective lens used in these cells (35 nm) demonstrated a clear advantage over the 60 nm optic for imaging smaller yeast cells, where the whole cell was imaged at high resolution. We also obtained better resolution information in the larger B cell with the 35 nm optic, but it clearly limited the field of view to a small region of the cell.

The glass capillary specimen holders used with the soft x-ray microscope has a varying diameter from 4 μm to 20 μm, holding an array of cell sizes and, with smaller cell types (e.g. yeast), multiple smaller cells in a field of view. To image this size variability and to extend the range of applications of soft x-ray microscopy even further, we envision continued upgrades of XM-2 to accommodate four objectives. For example, objectives with an 80 nm; 60 nm; 40 nm; 20 nm resolution range is particularly feasible with ongoing development of new iterative reconstruction schemes [45].

We also foresee the development of different acquisition schemes for soft x-ray microscopy to enable the imaging of large cells with high resolution, such as a combination of through-focus deconvolution [46–48] and tomographic acquisition approaches [49] or the computational combination of structural information from multiple resolutions [50, 51].

## Conclusion

In closing, the most comprehensive view of a cell is unlikely to come from a single microscope. Rather, it has become commonplace to combine data measured from the same specimen using a combination of disparate contrast mechanisms, typically differing in specimen illumination. SXT is no different. Much effort is now being put into combining SXT with other imaging methods, in particular cryogenic fluorescence microscopy. Having the ability to choose the SXT field of view and resolution will allow for better matches with fluorescence data collected from the same specimen, and ongoing developments will enable correlation with super resolution imaging technologies. The work and results presented here go a long way towards achieving this goal.
